# Correlation of Serum Asprosin Levels With Normalized Protein Catabolic Rate in Patients Receiving Peritoneal Dialysis Treatment

**DOI:** 10.7759/cureus.38441

**Published:** 2023-05-02

**Authors:** Sümeyra Koyuncu, Hilal Sipahioglu, Cihan Uysal, Cigdem Karakukcu

**Affiliations:** 1 Nephrology, Kayseri State Hospital, Kayseri, TUR; 2 Intensive Care Unit, Kayseri City Training and Research Hospital, Kayseri, TUR; 3 Nephrology, Erciyes University, Kayseri, TUR; 4 Biochemistry, Erciyes University, Kayseri, TUR

**Keywords:** asprosin, normalized protein catabolic rate (npcr), protein energy malnutrition, nutrition and metabolism, peritoneal dialysis (pd)

## Abstract

Background

Peritoneal dialysis patients are malnourished due to loss of protein in the dialysate and inadequate dialysis, although they take additional calories every day during treatment. Many parameters are used to assess nutritional status, with normalized protein catabolic rate (nPCR) being one of the most common. Asprosin, a novel adipokine secreted by adipose tissue, peaks during fasting and induces hepatic glucose release through the activation of the G-protein-cAMP-PKA pathway, which has been indicated to have a curative effect on chronic inflammation. In this study, we aimed to investigate the relationship between asprosin levels and nutritional parameters in patients receiving peritoneal dialysis treatment as well as to investigate the applicability of more practical tests.

Methodology

A total of 70 peritoneal dialysis patients, 35 female (59%) and 24 male (41%), were included in the study. The mean age of the patients was 53 ± 14 years (range = 18-80 years), and the median peritoneal dialysis duration was 31.5 months (range = 20-56.2 months). The most common etiologic cause was hypertension (37%). Patients over 18 years of age who had been receiving peritoneal dialysis treatment for at least 24 months were included in the study. The correlation between patients’ nPCR levels and serum asprosin, body mass index, and lipids was evaluated.

Results

The correlation between the level of nPCR and the serum asprosin level, body mass index, and lipids was evaluated. Patients with nPCR <0.815 were considered malnourished, and factors affecting malnutrition were determined by univariate analysis. Among the factors affecting malnutrition according to univariate analysis, those with p-value <0.05 were analyzed by multivariate analysis. Low asprosin level was one of the independent factors affecting malnutrition in patients (Exp(B) = 0.944, 95% confidence interval (CI) = 0.896-0.994). Other independent factors affecting malnutrition were Kt/V (Exp(B) = 0.018, 95% CI = 0.001-0.550) and residual renal function (Exp(B) = -0.004, 95% CI = 0.993-0.999).

Conclusions

There is a need for more accessible tests and reliable parameters to evaluate dialysis and nutritional deficiency in peritoneal dialysis patients. One possible hormone that could serve as a guide is asprosin.

## Introduction

Peritoneal dialysis is one of the two main renal replacement modalities for patients with end-stage renal failure. Although it is an effective treatment method, it is associated with various long-term complications [1]. Although consumption of 1.2-1.3 g/kg of protein is generally recommended, malnutrition can be observed in 30-50% of patients [2]. Peritoneal dialysis patients are malnourished due to loss of protein in the dialysate and inadequate dialysis, although they take additional calories every day during treatment.

Several parameters are used to assess nutritional status. Markers such as serum albumin, hemoglobin, transferrin, and body mass index (BMI) are influenced by several factors such as age, gender, inflammatory status, and liver function which are not sufficiently sensitive. Normalized protein catabolic rate (nPCR) more accurately reflects the protein intake and is used more often to assess nutritional status in dialysis patients with possible prognostic significance [3,4]. Several parameters indicate inadequate dialysis. Malnutritional status is also an indicator of dialysis failure and is usually defined as nPCR <0.8 g/kg/day [5,6].

Asprosin, a recently discovered hormone secreted from white adipose tissue, increases glucose and insulin release during fasting. It is also known to stimulate the hypothalamic feeding center, leading to the stimulation of appetite and fat storage [7]. Dialysis failure also causes malnutrition and increases mortality and morbidity [1].

In this study, we aimed to investigate the relationship between the level of asprosin and nutritional parameters in patients receiving peritoneal dialysis as well as to investigate the applicability of more practical tests.

## Materials and methods

This study included 80 patients who were followed up in our outpatient peritoneal dialysis (CAPD) clinic between March and September 2022. Patients over 18 years of age who had been receiving peritoneal dialysis treatment for at least 24 months were included in the study. Patients with active infection, malignancy. and peritonitis within the last three months were excluded. A total of 59 patients were included in this study. Ethics committee approval was obtained from Kayseri City Hospital (approval number: 522; date: 10.02.2022).

The retrospective data of the patients during two years of follow-up were reviewed. Albumin variability and episodes of peritonitis were recorded.

Laboratory measurements such as hemoglobin, leukocytes, ferritin, blood urea nitrogen, creatinine, sodium, uric acid, calcium, phosphorus, parathyroid hormone, total cholesterol, triglyceride, and albumin were performed and recorded. All patients included in the study were on a standard peritoneal dialysis treatment program (2,000 or 2,500 mL volume change, four or five times daily) and were CAPD patients. Demographic and clinical information including four-hour peritoneal equalization test (D/P Cr); renal, peritoneal, and weekly Kt/V; nPCR (calculated using the Randerson formula) [8,9]; peritoneal ultrafiltration; and residual renal function (RRF) were recorded for each patient. Inadequate dialysis was defined as weekly total Kt/V urea <1.70 according to Kidney Disease Outcome Quality Initiative (KDOQI) and International Society for Prenatal Diagnosis (ISPD) guidelines. Total weekly Kt/V urea was calculated by summing peritoneal Kt/V urea (calculated from 24-hour peritoneal dialysate) and renal Kt/V urea (calculated from 24-hour urine) according to the formula recommended in the KDOQI guidelines [3,9,10].

Blood was collected from the patient into a 4 cc biochemistry tube after obtaining patient consent along with the routine examinations performed at the time of presentation to the outpatient clinic. The blood was centrifuged at 3,000 rpm for five minutes, and 3 mL of serum supernatant was removed and collected in an Eppendorf tube. Serum samples were kept frozen at -80°C. Serum asprosin protein concentrations were analyzed using the enzyme-linked immunosorbent assay method (Cat. no: E4095HU).

Statistical analysis

Statistical analysis was performed using SPSS statistical program version 22 (IBM Corp., Armonk, NY, USA). The conformity of continuous variables to normal distribution was examined using the Shapiro-Wilk test. Continuous variables were presented as mean ± SD or median (interquartile range; IQR) values according to normal distribution. Categorical variables were expressed as number (%, percentage). Patients were divided into two groups, namely, malnourished and non-malnourished according to nPCR value <0.815. Differences in numerical variables between different groups were analyzed by the analysis of variance test. The chi-square test compared categorical variables with nominal variables. Patients with nPCR value <0.815 were considered malnourished, and factors affecting malnutrition were analyzed as univariate by logistic regression test.

Factors with p-values <0.05 according to univariate analysis were included in multivariate analysis. Variables with p-values <0.05 were considered independent risk factors for malnutrition.

The relationship between nPCR and the level of asprosin was investigated using Spearman’s correlation test. In all analyses, p-values <0.05 were considered statistically significant.

## Results

A total of 70 peritoneal dialysis patients, 35 female (59%) and 24 male (41%), were included in this study. The mean age of the patients was 53 ± 14 years (range = 18-80 years), and the median peritoneal dialysis duration was 31.5 months (range = 20-56.2 months). The most common etiologic causes were diabetes, hypertension, and glomerulonephritis. There were no patients with dialysis failure in our group. Demographic characteristics and laboratory parameters of the patients are shown in Table 1.

**Table 1:** Demographic, clinical, and laboratory parameters of the patients.

Variables (n = 59)	Mean ± SD, median (IQR), n (%)
Ages, years	53 ± 14
Gender male/female, n (%)	24/35 (41/59)
Hypertension, n (%)	22 (37%)
Diabetes mellitus, n (%)	14 (23%)
Glomerulonephritis, n (%)	10 (16%)
Other, n (%)	13 (22%)
Body mass index, kg/m^2^	26.5 ± 5.6
Dialysis time, months	31.5 (20–56.2)
24-hour urine volume, mL	450 (0–1000)
Total Kt/V	2.2 ± 0.58
Normalized protein catabolic rate, g/kg/day	0.77 ± 0.1
Parathyroid hormone, pg/mL	364 (225–535)
Blood urea nitrogen, mg/dL	50 ± 12.6
Creatinine, mg/dL	7.7 ± 2.3
High-density lipoprotein-cholesterol, mg/dL	45 ± 15
Triglyceride, mg/dL	166 (116–251)
Sodium, mmol/L	137 ± 4.6
Potassium, mmol/L	4.3 ± 0.6
Calcium, mg/dL	8.7 ± 0.9
Phosphorus, mg/dL	4.6 ± 1.3
Albumin, g/L	3.7 ± 0.4
Total protein, g/L	6.7 ± 0.6
Hemoglobin, g/L	11.3 ± 1.7
Asprosin, ng/dL	33.9 (24.5–49.2)

The correlation between the level of nPCR and the serum level of asprosin, BMI, and lipids was evaluated. Patients were divided into two groups, namely, nPCR <0.815 and nPCR >0.815. There were significant differences between the groups in terms of Kt/V, asprosin, and RRF. The asprosin level was higher in the group with nPCR >0.815 (p = 0.023).

No statistically significant difference was found between the groups in terms of BMI, diabetes mellitus, sodium, calcium, phosphorus, low-density lipoprotein, total cholesterol, total protein, and albumin levels (p > 0.05) (Table 2).

**Table 2:** Comparison of biochemical parameters by normalized protein catabolic rate (nPCR).

Variables	nPCR <0.815	nPCR >0.815	P-value
Asprosin, ng/dL	28.4 (19.9–45.4)	42 (28.9–58.3)	0.023
Body mass index, kg/m^2^	23.9 ± 5	28.8 ± 5	<0.001
Diabetes mellitus, %	9 (64)	5 (36)	0.284
Total Kt/V	2.1 ± 0.58	2.4 ± 0.5	0.037
Albumin, g/L	3.79 ± 0.37	3.8 ± 0.5	0.801
Total protein, g/L	6.8 ± 0.6	6.6 ± 0.6	0.412
High-density lipoprotein-cholesterol, mg/dL	43 ± 14	48 ± 17	0:188
Triglyceride, mg/dL	209 ± 149	226 ± 166	0.279
Phosphorus, mg/dL	4.5 ± 1.3	4.7 ± 1.3	0.559
Calcium, mg/dL	8.8 ± 0.9	8.7 ± 1	0.542
Sodium, mmol/L	136 ± 4.5	137 ± 4.8	0.228
Residual renal function, mL	50 (0–500)	675 (0–1,200)	0.013

Patients with nPCR <0.815 were considered malnourished, and factors affecting malnutrition were first determined by univariate analysis. Among the factors affecting malnutrition according to univariate analysis, those with p-values <0.05 were analyzed by multivariate analysis. Low asprosin level was one of the independent factors affecting malnutrition in patients (Exp(B) = 0.944, 95% confidence interval (CI) = 0.896-0.994). Other independent factors affecting malnutrition were Kt/V (Exp(B) = 0.018, 95% CI = 0.001-0.550) and residual renal function (Exp(B) = -0.004, 95% CI = 0.993-0.999) (Table 3).

**Table 3:** Multivariate analyses of possible predictors nPCR <0.815 g/kg/day. BMI: body mass index; RRF: residual renal function; CI: confidence interval; OR: odds ratio; nPCR: normalized protein catabolic rate

	Coefficient	OR	95% CI	P-value
Asprosin	-0.058	0.944	0.896-0.994	0.028
BMI	0.374	1.454	1.145-1.847	0.002
Total Kt/V	-4.003	0.018	0.001-0.550	0.021
RRF	-0.004	0.996	0.993-0.999	0.021

**Figure 1:**
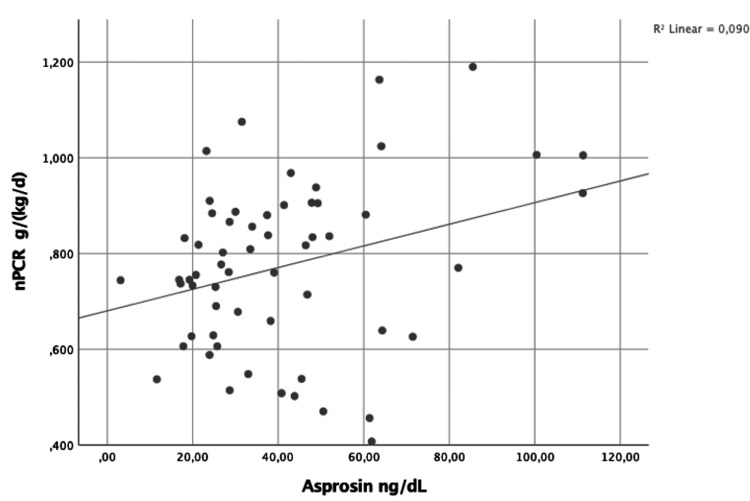
Asprosin and nPCR correlation (Ro = 0.299; p = 0.021) nPCR: normalized protein catabolic rate

**Figure 2:**
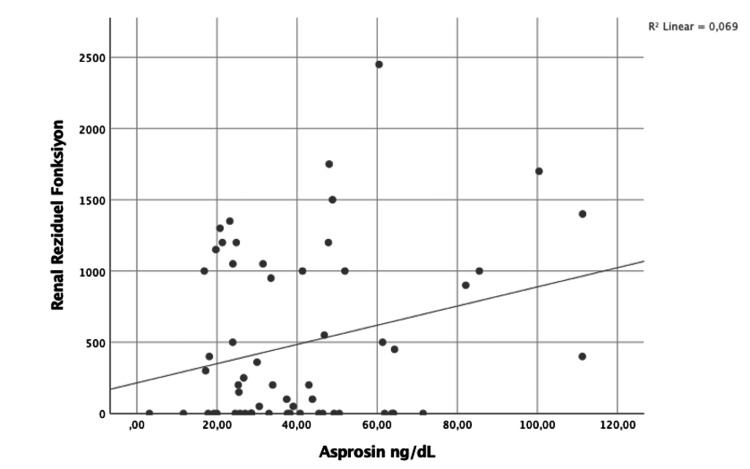
Asprosin and RRF correlation (Ro = 0.262; p = 0.045) RRF: residual renal function

There was a positive weak correlation between nPCR and asprosin (Ro = 0.299; p = 0.021) (Figure 1). Furthermore, there was also a positive weak correlation between RRF and asprosin (Ro = 0.262; p = 0.045) (Figure 2).

## Discussion

Inadequate dialysis and nutritional deficiencies in patients receiving peritoneal dialysis treatment cause high morbidity and mortality. This is usually assessed by total urea clearance [3,9,11]. There were no patients with dialysis failure in our study group. However, we found that asprosin levels were higher in patients with low nPCR levels. In addition, asprosin levels were correlated with Kt/V.

There are many parameters that assess nutritional status. nPCR reflects protein intake and is commonly used in dialysis patients [7]. Malnutrition is generally defined by the KDOQI guidelines and other authorities as nPCR <0.8 g/kg/day [5,6,12,13].

Adequate nutrition is vital for the success of both peritoneal and hemodialysis. Mortality in peritoneal dialysis patients can be estimated by measuring total body nitrogen, an indicator of protein intake. Furthermore, advanced age, chronic inflammation, and loss of RRD lead to inadequate protein intake and altered protein balance [14,15]. Dialysis adequacy is a very important concept for nutritional status, and other contributing factors include blood pressure and volume status, nutritional status, acid-base status, and mineral and bone pathologies. The correlation between nutritional status and dialysis adequacy was demonstrated by Hemayati et al. [16].

Malnutrition directly contributes to the development of infection, making peritoneal dialysis more difficult. We examined the rate of peritonitis and the relationship between asprosin and nPCR in our patients. However, we did not find a significant relationship because our peritonitis rates were low.

nPCR can also be used as an indicator of peritoneal insufficiency. A study revealed that gender (male), nPCR <0.815 g/kg/day, higher weight, and residual glomerular filtration rate <2.43 mL/minute/1.73 m^2^ were independent risk factors for inadequate dialysis. Among common nutritional markers, nPCR may be superior in predicting sorbent-assisted peritoneal dialysis adequacy [17].

The hormone asprosin is secreted from white adipose tissue and increases glucose and insulin release during fasting and is known to stimulate the hypothalamic feeding center. In a study among cancer patients, patients with anorexia had significantly lower levels of asprosin compared to those without anorexia. Therefore, asprosin can serve as a potential therapeutic target [18].

Studies have shown that the level of asprosin decreases with rapid weight loss in patients undergoing bariatric surgery [19,20]. In another study, it was correlated with nutritional adequacy in critically ill patients [21].

High asprosin levels have also been associated with insulin resistance and type 2 diabetes mellitus (T2DM). So far, numerous clinical studies have detected increased concentrations of plasma asprosin in prediabetic and T2DM patients [22-24]. These findings were independently associated with triglyceride levels, fasting blood glucose levels, and adiposity-related parameters. In a study by Li et al., the data were consistent with the previous study, but there was no association with plasma triglyceride levels [25]. In our study, we did not find any association between asprosin levels, diabetes, and lipid levels.

Serum albumin levels are a good indicator of nutrition and mortality; however, it is not a sufficient parameter to show dialysis insufficiency as there are peritoneal leaks in dialysis [1].

In addition, it has also been shown that asprosin contributes to wound healing, has protective effects on the myocardium, may ameliorate endothelial damage, and significantly improves left ventricular function [26,27]. Because peritoneal dialysis patients have an increased risk of coronary artery disease, asprosin may also have protective effects.

The study has certain limitations that need to be addressed. First, it is necessary to include a larger patient group to increase the statistical power of the results. Furthermore, anthropometric measurement methods should be employed to evaluate nutritional status indicators in greater detail, which would provide more accurate information. Additionally, controlling the level of asprosin in patients with dialysis failure would be a valuable approach to understanding its effects. However, it should be noted that there were no patients with dialysis failure in our unit, which limits the generalizability of our findings.

## Conclusions

There is a need for more accessible tests and reliable parameters to evaluate dialysis and nutritional deficiency in peritoneal dialysis patients. One possible hormone that could serve as a guide is asprosin.
